# Delhi-Triage and Treat Tuberculosis Initiative: An Important Step Towards Tuberculosis Elimination

**DOI:** 10.7759/cureus.50550

**Published:** 2023-12-15

**Authors:** Sankalp Yadav

**Affiliations:** 1 Medicine, Shri Madan Lal Khurana Chest Clinic, New Delhi, IND

**Keywords:** undernutrition, body mass index: bmi, tb, tuberculosis, triage

## Abstract

Tuberculosis is one of the oldest known infective diseases. It continues to pose a major threat to global health. In low- and middle-income countries, drug-resistant strains of tuberculosis have made the disease increasingly dangerous. In high-burden countries like India, people with tuberculosis may not always have a comprehensive examination for severe illness (requiring hospitalization management) due to oversaturated outpatient departments, inadequate diagnostic capacity, and a lack of manpower. Oftentimes, these patients are initiated on treatment, and detailed assessments for severe illnesses are missed. This is particularly important in a country with the greatest tuberculosis burden, where two tuberculosis-related deaths occur every three minutes. The present article throws light on this grave issue, emphasizing the need for early triage at the time of diagnosis, which would ultimately impact overall mortality and treatment outcomes.

## Editorial

Tuberculosis (TB) is still an immense threat to health in endemic nations. Drug-resistant strains of mycobacteria have made matters worse, mandating creative approaches for efficient treatment [1]. At the national level, according to the National TB Prevalence Survey (NATBPS) 2019-2021, the estimated point prevalence of microbiologically confirmed pulmonary tuberculosis among individuals over 15 years of age was 316 per lakh population. It was predicted that there were 312 cases of all forms of tuberculosis per lakh people. Overall, in the year 2022, out of the total TB cases notified, 14,71,190 (61%) were male, 9,48,190 (39%) were female, and 1,023 (<1%) belonged to lesbian, gay, bisexual, transgender, queer, intersex, asexual, and other identities [2].

As per the World Health Organization's (WHO) recently released Global TB Report 2023, India contributes 27% of the total cases of tuberculosis, with 22% mortality worldwide. India seeks to eliminate tuberculosis by the year 2025. The goal of the National Strategic Plan 2017-2025 was to achieve 44 new cases of tuberculosis per lakh of citizens by the end of 2025. This figure, according to the 2023 report, is 199 cases per lakh. Hitting this goal will prove difficult because, by 2023, the plan called for an incidence of only 77 cases per lakh population. By 2025, the program also seeks to lower mortality to three deaths per lakh of people. The WHO has acknowledged the updated numbers for India; yet, this still comes out to 23 per lakh people [3].

With the current data on significant mortality in tuberculosis, it becomes important to assess the determinants for the same. One of the factors was an improper assessment of the severity of the disease at the time of diagnosis. In a study by Shewade et al., out of 3,010 cases of tuberculosis, 1,529 (50.8%) were screened at the time of diagnosis or notification, of whom 537 (35.1%) had a high risk of severe illness. Further, when comparing individuals without a high risk of severe illness (3.8%) to those with a high risk of severe illness (8.9%) at diagnosis, the incidence of early fatalities was considerably greater. Furthermore, early mortality was highest in the first two weeks of the disease and was highly correlated with a high risk of severe illness upon diagnosis or notification [4].

The burden of severe disease at notification or diagnosis and the viability of gathering these data in standard program settings are topics that have received little attention in the literature. The National Tuberculosis Elimination Program guidelines for 2021 in India, unequivocally advise conducting a severity evaluation as soon as feasible upon diagnosis and referring patients for inpatient care if they are very sick. However, this guideline needs clinical, laboratory, and radiographic examinations, which might be difficult in peripheral health institutions [4]. To address the issues in severity evaluation in the National Tuberculosis Elimination Program guidelines for 2021, a 'Differentiated TB Care Model' was introduced, which was basically a simplified approach with criteria for hospitalization [5].

As recommended from 16 districts of Karnataka in the findings of the study by Shewade et al., an easy and quick method for early assessment of the severity of illness based on vital signs, body mass index (BMI), and the inability to stand without assistance are simple ways to screen for the severity of the illness. These indicators are well-known risk factors for death that are straightforward to evaluate and understand. Patients who exhibit any of these signs may be directed to more advanced facilities, like a tertiary care hospital, for inpatient care and an extensive clinical evaluation [4].

A similar initiative is planned for Delhi, involving all 25 chest clinics or district tuberculosis centers. Beginning January 1, 2024, all adults (more than or equal to 15 years of age) who will be diagnosed with tuberculosis, both drug-sensitive and drug-resistant, notified by public health institutes will be triaged. This activity will be started as a pilot project on December 15, 2023. This triage will be done by assessing five indicators (Table 1).

**Table 1:** Five indicators for assessment of the severity of illness. BMI: Body mass index. Reference [4].

Indicator	Assessment
BMI (1)	Less than or equal to 14.0 kg/m^2^
BMI (2)	14.1 to 16.0 kg/m^2 ^with swelling in the legs
Respiratory rate (3)	More than 24/minute
Oxygen saturation (4)	Less than 94% on room air
Ability to stand (5)	Without support, i.e., standing with support/squatting/sitting/bedridden

This triaging will be done as soon as possible at the diagnosing facility without even waiting for a formal notification of the patient or at the next earliest opportunity (at the home visit or start of the treatment or at the time of baseline investigations for tuberculosis). Two categories for severity of illness are defined based on the triage: either a triage positive or a triage negative. All the cases marked as triage positive will be registered in the notification registers at the health facility diagnosing the case and referred immediately to the designated tertiary care hospital in the national capital of India. Two hospitals, the National Institute of Tuberculosis and Respiratory Diseases and the Rajan Babu Institute of Pulmonary Medicine and Tuberculosis, are part of this initiative where these referrals will be made and a specific number of beds with trained staff designated to take care of these severe cases are planned. The five indicators mentioned in Table 1 would help in detecting three conditions at the time of diagnosis (Table 2).

**Table 2:** Conditions were detected based on the presence of indicators. Reference [4].

Presence of indicator	Condition detected
1 or 2	Very severe undernutrition
3 or 4	Respiratory insufficiency
5	Poor performance status

In India and other high-burden nations, there is a paucity of data on screening for severe illness on diagnosis or notification and the correlation between severe illness and early tuberculosis death in programmatic settings [4]. In a large study from Andhra Pradesh by Jonnalagada et al. on 8,240 tuberculosis patients, 50% of early deaths were reported within the first four weeks of treatment [6]. This data rose to 75% in the study from Karnataka [4]. Besides, another study from the Indian state of Tamil Nadu (during April-June 2022) implemented a differentiated care strategy called Tamil Nadu-Kasanoi Erappila Thittam (TN-KET) for all adults aged 15 years and older with drug-susceptible tuberculosis notified by public health facilities. 

Following notification of 14,961 TB patients, 11,599 (78%) underwent triage. Out of the people who were triaged, 1,509 (13%) had a high risk of developing a severe illness; of these, 1,128 (75%) underwent a thorough clinical evaluation in a nodal inpatient care health facility. In this study, 909 (92%) out of 993 confirmed as severely ill, were admitted, with 8% unfavorable admission outcomes, including 4% deaths [7].

To conclude, initial screening for severe sickness as an alternative strategy to reduce tuberculosis fatalities has existed since 2021, but a quicker and more targeted approach considering the issues at the grassroots level is warranted. This initiative in Delhi would be a remarkable step toward reducing mortality due to tuberculosis and achieving the desired aims of the National Strategic Plan 2017-2025. Additionally, this is the first such initiative where both drug-sensitive and drug-resistant tuberculosis cases will be triaged. 
